# Controlling synchronization of gamma oscillations by astrocytic modulation in a model hippocampal neural network

**DOI:** 10.1038/s41598-022-10649-3

**Published:** 2022-04-28

**Authors:** Sergey Makovkin, Evgeny Kozinov, Mikhail Ivanchenko, Susanna Gordleeva

**Affiliations:** 1 Department of Applied Mathematics, Institute of Information Technology, Mathematics and Mechanics, Lobachevsky University, 23, Gagarin Ave., Nizhny Novgorod, 603022 Russia; 2 Department of Supercomputer Computation, Institute of Information Technology, Mathematics and Mechanics, Lobachevsky University, 23, Gagarin Ave., Nizhny Novgorod, 603022 Russia; 3 Neurotechnology Department, Institute of Biology and Biomedicine, Lobachevsky University, 23, Gagarin Ave., Nizhny Novgorod, 603022 Russia; 4 Neuroscience and Cognitive Technology Laboratory, Center for Technologies in Robotics and Mechatronics Components, Innopolis University, 1, Universitetskaya Str., Innopolis, 420500 Russia; 5 Center for Neurotechnology and Machine Learning, Immanuel Kant Baltic Federal University, 14, Nevskogo Str., Kaliningrad, 236041 Russia

**Keywords:** Biophysical models, Complex networks, Applied mathematics

## Abstract

Recent in vitro and in vivo experiments demonstrate that astrocytes participate in the maintenance of cortical gamma oscillations and recognition memory. However, the mathematical understanding of the underlying dynamical mechanisms remains largely incomplete. Here we investigate how the interplay of slow modulatory astrocytic signaling with fast synaptic transmission controls coherent oscillations in the network of hippocampal interneurons that receive inputs from pyramidal cells. We show that the astrocytic regulation of signal transmission between neurons improves the firing synchrony and extends the region of coherent oscillations in the biologically relevant values of synaptic conductance. Astrocyte-mediated potentiation of inhibitory synaptic transmission markedly enhances the coherence of network oscillations over a broad range of model parameters. Astrocytic regulation of excitatory synaptic input improves the robustness of interneuron network gamma oscillations induced by physiologically relevant excitatory model drive. These findings suggest a mechanism, by which the astrocytes become involved in cognitive function and information processing through modulating fast neural network dynamics.

## Introduction

Synchronous rhythms of the brain support a variety of cognitive functions by providing temporal and spatial coordination of neural network signaling^1,2^. Particularly, fast gamma cortical oscillations ($\gamma$ 20–80 Hz) have been recorded in many cortical brain structures during both waking and sleep states. They are commonly associated with sensory processes^3^, attention^4^, learning, memory storage, and retrieval^5,6^. Nonetheless, functions of $\gamma$ oscillations, their cellular and network mechanisms remain a matter of debate^7^. Experimental and theoretical evidence suggests that the generation of $\gamma$ network oscillations critically depends on the rhythmic activity of local networks of synaptically connected GABAergic interneurons, which synchronize spikes in pyramidal neurons^8–12^. Although the key contribution of interneurons to $\gamma$ rhythm formation has been well established, the mechanisms underlying the generation of coherent oscillations in the interneuron networks have not been fully clarified yet.

Following the experimental findings, which show that interneurons, in particular fast-spiking basket cells (BCs), can generate $\gamma$ activity in vitro^8,10^ and in vivo^13,14^, numerous computational studies proposed mechanisms of synchrony emergence in a recurrent network of interneurons in response to a tonic excitatory drive^11,15–18^. A major challenge of interneuron network models is understanding the mechanisms of the robustness of gamma oscillations against heterogeneity in the excitatory drive (for review, see^19^). Existing models with slow, weak, and hyperpolarizing synapses generate synchronized gamma activity only when the heterogeneity of the input drive is low and the amplitude is high^11^, which is physiologically inconsistent^20,21^. It was shown that sensitivity to heterogeneity can be significantly reduced by incorporating realistic synaptic properties in the models^12,15,22^. Search for the biologically plausible mechanisms to improve the robustness of coherent oscillation formation in the interneuron network models against heterogeneity remains a highly relevant task.

Recently, it was shown that glial cells substantially influence the formation of gamma oscillatory rhythms, which were previously thought to be a product of neuronal activity^23–25^. In particular, it was revealed that astrocytic vesicular release is necessary to maintain functional gamma oscillations, and it is essential for novel object recognition behavior both in vitro and in awake-behaving animals^23^. However, the cellular and network mechanisms underlying astrocytic involvement in the formation of synchronized neural network $\gamma$ activity remain undefined.

Astrocytes sense and integrate neuronal activity by responding with intracellular Ca$^{2+}$ elevations^26^. Due to the fact that astrocytic calcium dynamics has a significantly slower timescale than the fast synaptic transmission of neurons, it has been assumed for decades that astrocytes do not play a major role in modulating neural network activity, neural information processing, and cognition. Nevertheless, recent studies have shown that astrocytes can regulate neuronal excitability, synaptic transmission, and plasticity^27,28^ through the release of chemical transmitters (termed “gliotransmitters”) induced by intracellular Ca$^{2+}$ signals. Although these findings have been independently confirmed by several groups, the functional significance and properties of gliotransmission remain a matter of debate^29,30^. Numerous studies reveal the unexpected role of astrocytes in the coordination of the fast dynamics of neural circuits that underlie normal cognitive behaviors^31–33^. Several computational studies discuss the role of astrocytes in the spatio-temporal coordination of neural network signaling^34–39^, the emergence of coherent oscillations^40,41^, information processing^42–46^, and memory formation^47–51^. Savtchenko and Rusakov showed that the astroglia-like, volume-limited synaptic regulation of excitatory input appears to be better at preserving interneuron network synchronization while inducing the network clustering to neuron subgroups with distinct firing patterns^41^.

Nonetheless, the way the interplay of slow modulatory astrocytic signaling with fast synaptic transmission is involved in the formation of coherent oscillations in the interneuron network remains an open question. To address this issue, we studied the role of astrocyte-mediated regulation of synaptic transmission in the generation of $\gamma$ interneuron network oscillations. In particular, we investigated the computational model of hippocampal interneuron network which displays physiologically plausible oscillatory behaviors^11,12,17^. To do that we complemented it with an astrocytic network and explored how $\gamma$ oscillations in the interneuron network can be regulated by the astrocytic modulation of synaptic transmission.

## Methods

In our previous work, we investigated the dynamics of the bidirectional neuron-astrocyte interaction in a minimal network model^36^. We showed that astrocytes can induce the intermittent synchronization of a pair of synaptically coupled fast spiking neurons on the slow timescale of calcium oscillations. Here, we employ a similar approach to study the dynamics of an extended version of the model .

**Figure 1:**
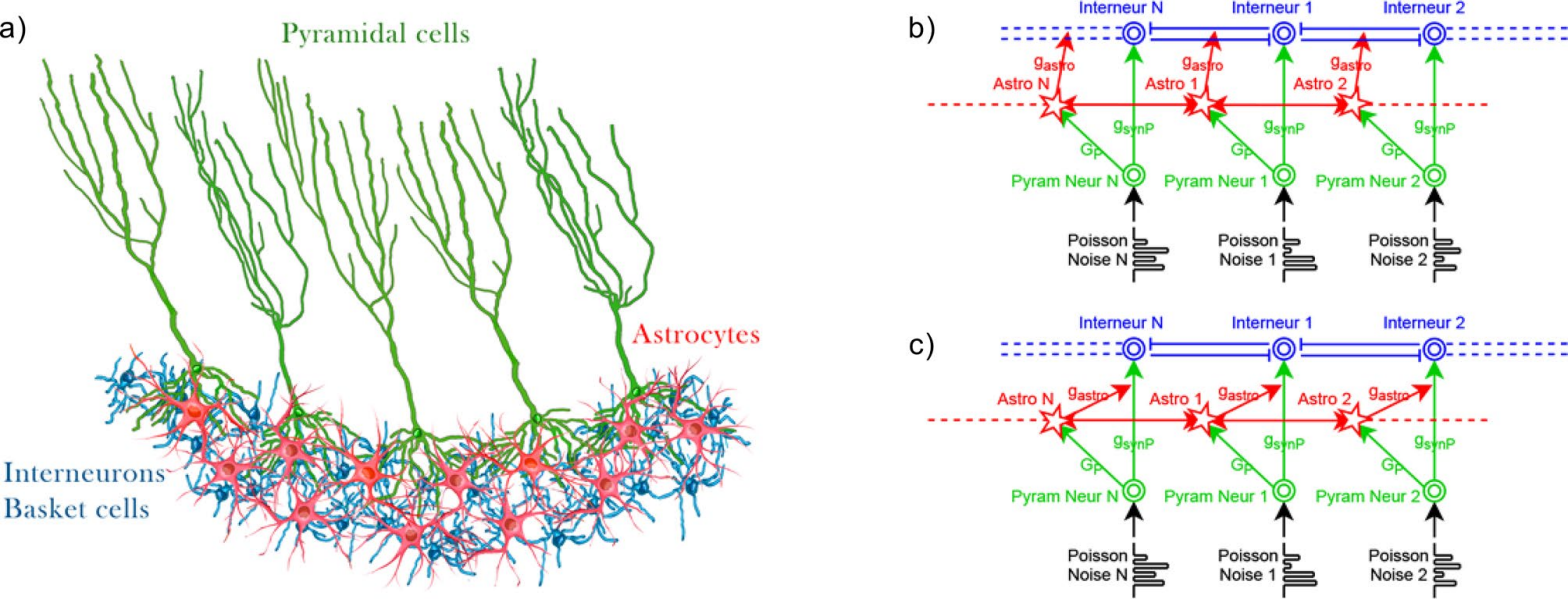
(**a**) A scheme illustrating the organization of modeled BCs-pyramidal cells-astrocytes network in hippocampal area CA1^55^. (**b**,**c**) Architecture of the neuron-astrocyte network model. The interneuron network model consists of 200 neurons arranged on a virtual ring^15^, which mimics the organization of the basket cell network. Each neuron is randomly connected to its 100 nearest neighbors by inhibitory chemical synapses with a probability of 0.5. Interneurons receive excitatory inputs from pyramidal neurons whose stochastic firing is triggered by the random Poisson spike trains. Astrocytes are organized in a ring-shaped network repeating the topology of the interneuron network. Each astrocyte in the proposed model is connected to the neighboring astrocytes through gap junctions. Two types of astrocytic influence on signal transmission in the neuronal network are considered: astrocyte-induced modulation of (**b**) inhibitory synaptic transmission in the interneuron network and (**c**) excitatory synaptic transmission from pyramidal neurons to interneurons.

We studied the interneuron network model of 200 neurons arranged on a virtual ring^15^, which mimicked the organization of the BC network in hippocampal area CA1. Each neuron was randomly connected to its 100 nearest neighbors by inhibitory chemical synapses with a probability of 0.5. This connectivity reflects anatomical analyses of functional links among interneurons in area CA1^52^. Interneurons receive excitatory inputs from pyramidal neurons whose stochastic firing is triggered by the random Poisson spike trains with a given rate. Astrocytes are organized in a ring-shaped network repeating the topology of the interneuron network. It was shown that the number of astrocytes and neurons is approximately the same in the mammalian brain and that the individual astrocytes occupy separate, non-overlapping tissue domains^53^. The astrocytic network in the proposed model consists of 200 diffusely coupled astrocytes. Each astrocyte is connected to the two nearest neighboring astrocytes through gap junctions^54^. Astrocytes generate the elevations of intracellular Ca$^{2+}$ in response to a synaptically released neurotransmitter (glutamate) from the pyramidal neurons. Such Ca$^{2+}$ activity can regulate the strength of synaptic connections of near and distant tripartite synapses at diverse timescales through gliotransmitter release^27^. It was shown that locations of dendritic trees of the BCs in the hippocampus do not significantly overlap with each other^55^. Therefore, an individual astrocyte can influence only one or a small proportion of BCs from the entire network^41^. The architecture of the simulated neuron-astrocyte network is shown in Fig. 1. Each astrocyte is coupled to one corresponding interneuron and acts by modulating incoming connections of the neuron from interneurons or from pyramidal neuron. In this paper, we studied two types of astrocytic modulations of signal transmission in the interneuron network. In particular, we considered astrocyte-induced modulation of (i) inhibitory synaptic transmission in an interneuron network (Fig. 1b) and (ii) excitatory synaptic transmission from pyramidal neurons to interneurons (Fig. 1c).

### Neural network

Various models describe the neuronal spiking dynamics at different levels of bio-fidelity, from simplified leaky integrate-and-fire model to biophysical Hodgkin-Huxley equations^56^. Here we use the Mainen modification of the Hodgkin-Huxley neuron model for the mammalian brain^57,58^, as the most biologically plausible. Membrane potential neuronal dynamics was modeled as

1 $$\begin{aligned} \left\{ \begin{aligned} &{} \frac{{{d}\ \!{V}}_i}{{{d}{t}}} = \frac{1}{C} \left( {I_{channel}}_i + {I_{app}} + {I_{P}}_i +{I_{syn}}_i \right) ,\vspace*{6.25pt}\\ &{} \frac{{{d}\ \!{x}}_i}{{{d}{t}}} = \alpha _x (1 - x_i) - \beta _x x_i , \; x = m, n, h ;\\ &{}{I_{channel}}_i= g_{Na} m_i^3 h_i (E_{Na} - V_i) + g_K n_i (E_K - V_i) + g_{Leak} (E_{Leak} - V_i), \\ \end{aligned} \right.  \end{aligned}$$

where *i* $(i = 1,\ldots , 200)$ corresponds to a neuronal index, the transmembrane potential *V* is given in mV, and time *t* in ms. $I_{channel}$ is the sum of the transmembrane ionic currents (i.e., sodium, potassium, and leak currents). Nonlinear functions $\alpha _x$ and $\beta _x$ for gating variables are taken from the Mainen modification of the Hodgkin–Huxley model^57,58^:

2 $$\begin{aligned} & \alpha _m = \frac{0.182 (V_i + 35)}{1 - e^{\frac{-(V_i + 35)}{9}}} ;&\beta _m = \frac{-0.124 (V_i + 35)}{1 - e^{\frac{(V_i + 35)}{9}}} ;\\&\alpha _n = \frac{0.02 (V_i - 25)}{1 - e^{\frac{-(V_i - 25)}{9}}} ;&\beta _n = \frac{-0.002 (V_i - 25)}{1 - e^{\frac{(V_i - 25)}{9}}} ;\\&\alpha _h = 0.25 e^{\frac{-(V_i + 90)}{12}} ;&\beta _h = 0.25 \frac{e^{\frac{V_i + 62}{6}}}{e^{\frac{V_i + 90}{12}}} .\\ \end{aligned}$$

Throughout this paper, we use the following parameter values: $C = 1\ \upmu{\text{F}}/{\text{cm}}^2$; $g_{Na} = 40\ {\text{mS}}/{\text{cm}}^2$; $g_K = 35\ {\text{mS}}/{\text{cm}}^2$; $g_{Leak} = 0.3\ {\text{mS}}/{\text{cm}}^2$; $E_{Na} = 55\ {\text{mV}}$; $E_K = -\,77\ {\text{mV}}$; $E_{Leak} = -\,54.4\ {\text{mV}}$. The constant applied current $I_{app}$ defines the dynamical regime (oscillatory, bistable, or excitable) of a neuron^59,60^. We chose the rest mode of a neuron with an equilibrium state of steady focus with a shift current value $I_{app}=0.7\ \upmu{\text{A}}/{\text{cm}}^2$^36^.

Each pyramidal neuron receives an external input $I_{P}$ defined as a Poisson pulse train with mean rate $F_{in}$ of 0–400 Hz chosen in accordance with earlier estimates^41^. The pulse has a rectangular shape with a constant duration of $2\ \text{ms}$, and amplitudes sampled independently from a uniform distribution in $[0.0;\ 2.5]\ \upmu{\text{A}}/{\text{cm}}^2$. Each pulse train is generated independently for each neuron. For interneurons $I_{P} = 0$.

The total synaptic current $I_{syn}$ received by neuron *i* from *N* presynaptic neurons is defined as^42,59^:

3 $$\begin{aligned} & {I_{syn}}_i = \sum _j^{N} \frac{\tilde{g}_{syn} ( V_i - {E_{syn}}_j)}{1 + e^{\frac{-V_j}{k_{syn}}}} ;\\&\tilde{g}_{syn} = {\left\{ \begin{array}{ll} g_{syn} (1 + g_{astro} [Ca^{2+}]_i),\;\; &{}\text{ if }\;[Ca^{2+}]_i \ge 0.3\;\upmu{\text{M}}, \\ g_{syn},\;\;&{}\text{ otherwise } \end{array}\right. } \\ \end{aligned}$$

where *j* denotes a presynaptic neuronal index, the reversal potential is $E_{syn} = -\,90\ {\text{mV}}$ for the inhibitory synapse and $E_{syn} = 0\ {\text{mV}}$ for the excitatory one, $k_{syn} = 0.2\ {\text{mV}}$. Parameter $\tilde{g}_{syn}$ corresponds to the synaptic weight and incorporates the astrocyte modulation due to the release of gliotransmitters in the tripartite synapses. To describe the astrocytic impact in synaptic transmission, we use a simplified approach tested in our previous studies^36,42,44^. We assume that upon reaching the intracellular calcium concentration, [Ca$^{2+}$], the threshold in $0.3\ \upmu{\text{M}}$, astrocytes release the gliotransmitters to the synaptic cleft whose interaction with pre- and postsynaptic terminals regulates the strength of the synaptic connections. Parameter $g_{syn}$ is the baseline synaptic weight in neuron-neuron communication (we use $g_{syn}$ to denote the synaptic weight between interneurons and $g_{syn_P}$ for the synaptic weight between pyramidal neurons and interneurons). Parameter $g_{astro}$ is the strength of the astrocyte-induced modulation of the synaptic weight.

### Astrocytic network

Each astrocyte tracks the activity of pyramidal neurons and can generate the elevation of the intracellular concentration of IP$_3$ followed by the emergence of calcium pulse in response to changes in neurotransmitter (glutamate) concentration in the synaptic cleft. When the pyramidal neuron *i* generates an action potential, it causes the release of glutamate from the synapse. The glutamate concentration is described by the following equation^36,44,61^:

4 $$\begin{aligned} & \frac{{{d}{G}}_i}{{{d}{t}}} = -{\alpha _G} {G}_i + {\beta _G} \frac{1}{1 + e^{(- \frac{{V}_i}{0.5})}}, \\ \end{aligned}$$

where $\alpha _G = 25\ {\text{s}}^{-1}$ and $\beta _G = 500\ {\text{s}}^{-1}$ denote the relaxation and production rates of glutamate. Glutamate binds to the metabotropic glutamate receptors on the astrocytic membrane, which is located close to the synapse and activates IP$_3$ signaling in the astrocyte. The dynamics of the intracellular concentration of IP$_3$ in astrocytes is expressed as follows^62^:

5 $$\begin{aligned} & \frac{{{d}{I}}P3_i}{{{d}{t}}} = \frac{{IP3}^* - IP3_i}{\tau _{IP3}} + {J_{PLC}}_i + {{J_{IP3}}_{diff}}_i + {J_{Glu}}_i, \\&{J_{PLC}}_i = v_4 \left( [Ca^{2+}]_i + (1 - \alpha ) k_4 \right) / ([Ca^{2+}]_i + k_4),\\&{{J_{IP3}}_{diff}}_i = d_{IP3} (IP3_{i-1} + IP3_{i+1} - 2 IP3_i) ;\\ \end{aligned}$$

where the parameter ${IP3}^*$ is the steady-state intracellular concentration of IP$_3$, currents $J_{PLC}$, ${J_{IP3}}_{diff}$ denote the IP$_3$ production caused by the activation of PLC by the calcium released from the endoplasmic reticulum, and the IP$_3$ diffusion between neighboring cells through the gap junctions, respectively. The current $J_{Glu}$ describes the glutamate-induced production of the IP$_3$ in response to neuronal activity and is modeled as^36,44^

6 $$\begin{aligned} & {J_{Glu}}_i = \frac{\alpha _{Glu}}{1 + e^{\frac{-({G}_i - 0.25)}{0.01}}} .\\ \end{aligned}$$

Glutamate released from presynaptic neurons is integrated over a larger timescale by the IP$_3$ dynamics through $J_{Glu}$. An increase in cytosolic IP$_3$ induces the opening of the Ca$^{2+}$-dependent receptors at the endoplasmic reticulum (ER), which results in a Ca$^{2+}$ influx from ER to the cytosolic volume. This process is known as calcium-induced calcium release (CICR) and can be described by the widely used biophysical model for astrocytic dynamics^62^:

7 $$\begin{aligned} {\left\{ \begin{array}{ll} \begin{aligned} &{} \frac{{{d}{[}}Ca^{2+}]_i}{{{d}{t}}} = {J_{ER}}_i - {J_{pump}}_i + {J_{leak}}_i + {J_{in}}_i - {J_{out}}_i + {{J_{Ca}}_{diff}}_i, \\ &{} \frac{{{d}{z}}_i}{{{d}{t}}} = a_2 \left( d_2 \frac{IP3_i + d_1}{IP3_i + d_3} (1 - z_i) - [Ca^{2+}]_i z_i \right) , \\ \end{aligned} \end{array}\right. } \end{aligned}$$

where $[Ca^{2+}]$ is the intracellular concentration of $Ca^{2+}$, and *z* is the fraction of IP$_3$ receptors that have not been inactivated by Ca$^{2+}$. $J_{ER},\ J_{pump},\ J_{leak}$ denote the fluxes from ER to the cytosol by the joint gating of Ca$^{2+}$ and IP$_3$, via the ATP-dependent pump from the cytosol to the ER, and the leaked flux from the ER to the cytosol, respectively. Calcium exchange with the extracellular space is described by $J_{in}$ and $J_{out}$. The flux ${J_{Ca}}_{diff}$ is the diffusive flux of Ca$^{2+}$ between astrocytes via gap junctions^63^. They evolve according to the following equations^62^:

8 $$\begin{aligned} & {J_{ER}}_i = c_1 v_1 IP3_i^3 [Ca^{2+}]_i^3 z_i^3 \left( \frac{c_0}{c_1} - (1 + \frac{1}{c_1}) [Ca^{2+}]_i \right) / \left[ (IP3_i + d_1)([Ca^{2+}]_i + d_5) \right] ^3 ;\\&{J_{pump}}_i = v_3 [Ca^{2+}]_i^2 / (k_3^2 + [Ca^{2+}]_i^2) ;\\&{J_{leak}}_i = c_1 v_2 \left( \frac{c_0}{c_1} - (1 + \frac{1}{c_1}) [Ca^{2+}]_i \right) ;\\&{J_{in}}_i = v_5 + v_6 IP3_i^2 / (k_2^2 + IP3_i^2) ;\\&{J_{out}}_i = k_1 [Ca^{2+}]_i ;\\&{{J_{Ca}}_{diff}}_i = d_{Ca} ([Ca^{2+}]_{i-1} + [Ca^{2+}]_{i+1} - 2 [Ca^{2+}]_i) .\\ \end{aligned}$$

Biophysical meaning of all parameters in Eqs. (5–8) and their experimentally determined values can be found in^62,64^. For our purposes, we fix $c_0 = 2\ \upmu{\text{M}}$; $c_1 = 0.185$; $v_1 = 6\ {\text{s}}^{-1}$; $v_2 = 0.11\ {\text{s}}^{-1}$; $v_3 = 2.2\ \upmu{\text{M}}/{\text{s}}$; $v_4 = 0.3\ \upmu{\text{M}}/{\text{s}}$; $v_5 = 0.025\ \upmu{\text{M}}/{\text{s}}$; $v_6 = 0.2\ \upmu{\text{M}}/{\text{s}}$; $k_1 = 0.5\ {\text{s}}^{-1}$; $k_2 = 1\ \upmu{\text{M}}$; $k_3 = 0.1$; $k_4 = 1.1\ \upmu{\text{M}}/{\text{s}}$; $a_2 = 0.14\ \upmu{\text{M}}/{\text{s}}$; $d_1 = 0.13\ \upmu{\text{M}}$; $d_2 = 1.049\ \upmu{\text{M}}$; $d_3 = 0.9434\ \upmu{\text{M}}$; $d_5 = 0.082\ \upmu{\text{M}}$; $\alpha = 0.8$; $\tau _{IP3} = 7.143\ {\text{s}}$; $IP3^* = 0.16\ \upmu{\text{M}}$; $d_{Ca} = 0.001\ {\text{s}}^{-1}$; $d_{IP3} = 0.12\ {\text{s}}^{-1}$; $\alpha _{Glu} = 2$. We rescale the time units of the astrocyte model in order to match it in milliseconds for numerical integration.

### Numerical methods

Simulations were done using a finite difference integration scheme based on the fourth-order Runge-Kutta algorithm with time step $\Delta t = 5 \cdot 10^{-3}$ ms. The total simulation time was 180 s. Network signaling was analyzed in the time interval from 15 to 180 s. Initial conditions used for simulation can be found in Appendix A (Supplementary Information).

### A measure of network coherence

Frequency and coherence of interneuron network activity were determined in 500-ms epochs. Average firing frequency, Ω, was determined as the inverse of the mean interspike interval. Neuronal membrane potentials were binarized (0—no action potential, 1—action potentials were generated in a given time interval). The network coherence was calculated as the mean of the coherence in all pairs of interneurons in the time window $\tau = 0.1 /{\Omega}$, $k(\tau )$, and was defined as the following^11,12^:

9 $$\begin{aligned} k_{i, j}(\tau ) = \frac{\sum ^L_{l=1} X(l) Y(l)}{\sqrt{\sum ^L_{l=1} X(l) \sum ^L_{l=1} Y(l)}}, \end{aligned}$$

where *i* and *j* denote the two interneurons, *X*(*l*) and *Y*(*l*) are the binary action potential patterns, and *L* is the number of time bins. In the case of full synchrony, $k(\tau )$ is 1 for all nonzero $\tau$ values; whereas in the case of total asynchrony, $k(\tau )$ becomes a linearly increasing function of $\tau$. To measure the network coherence in the course of astrocyte-mediated modulation of synaptic transmission, $k_{astro}$, we calculate the extrema of the coherence, *k*, during the Ca$^{2+}$ elevations in astrocytes ([Ca$^{2+}$] > 0.3 μM), $k_{astro_i}$, and then average them over all calcium pulses in the simulation.

## Results

In situ and in vivo experimental studies reveal a rich diversity of physiological consequences of astrocyte-mediated neuromodulation^27^. Involved in the same neuronal circuit astrocytes can release various types of gliotransmitters that can modulate synaptic transmission in different ways. For instance, in the hippocampus, the astroglial release of glutamate can potentiate inhibitory transmission by triggering presynaptic kainate receptors^65^ and support neuronal synchrony by triggering postsynaptic NMDARs^66^. In the hippocampal CA1 region, the gliotransmitter ATP can also depress or enhance excitatory synaptic transmission by triggering either A1 or A2 receptors, respectively^67,68^. To mimic multiple forms of astrocytic physiological actions on synaptic transmission in the modeled network, we explored two scenarios of neuron-astrocyte interaction with variable gliotransmitter-induced changes in synaptic transmission. We explored how synchrony in an interneuron network can be regulated by the astrocytic influence (i) on inhibitory transmission in the interneuron network (Fig. 1a), and (ii) on excitatory synaptic inputs from pyramidal neurons to interneurons (Fig. 1b).

**Figure 2:**
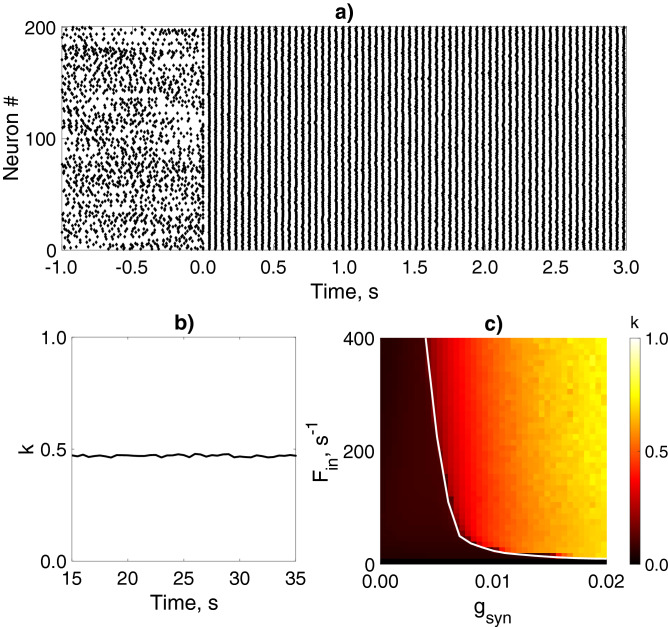
$\gamma$ oscillations in the interneuron network model. (**a**) Raster plots of the interneuron network activity with default parameter settings. Synapses were activated at 0 s ($g_{syn}=0.01$ mS/cm^2^, $F_{in} = 260\ {\text{s}}^{-1}$). (**b**) Coherence (*k*) is determined in 500-ms windows for the network oscillations shown on (**a**). (**c**) Mean network coherence (*k*) is plotted against the frequency of excitatory inputs ($F_{in}$) and the synaptic weight between interneurons ($g_{syn}$). The white line corresponds to the border of the synchronization region with $k = 0.4$. Each pixel represents an average of over 10 simulations.

**Figure 3:**
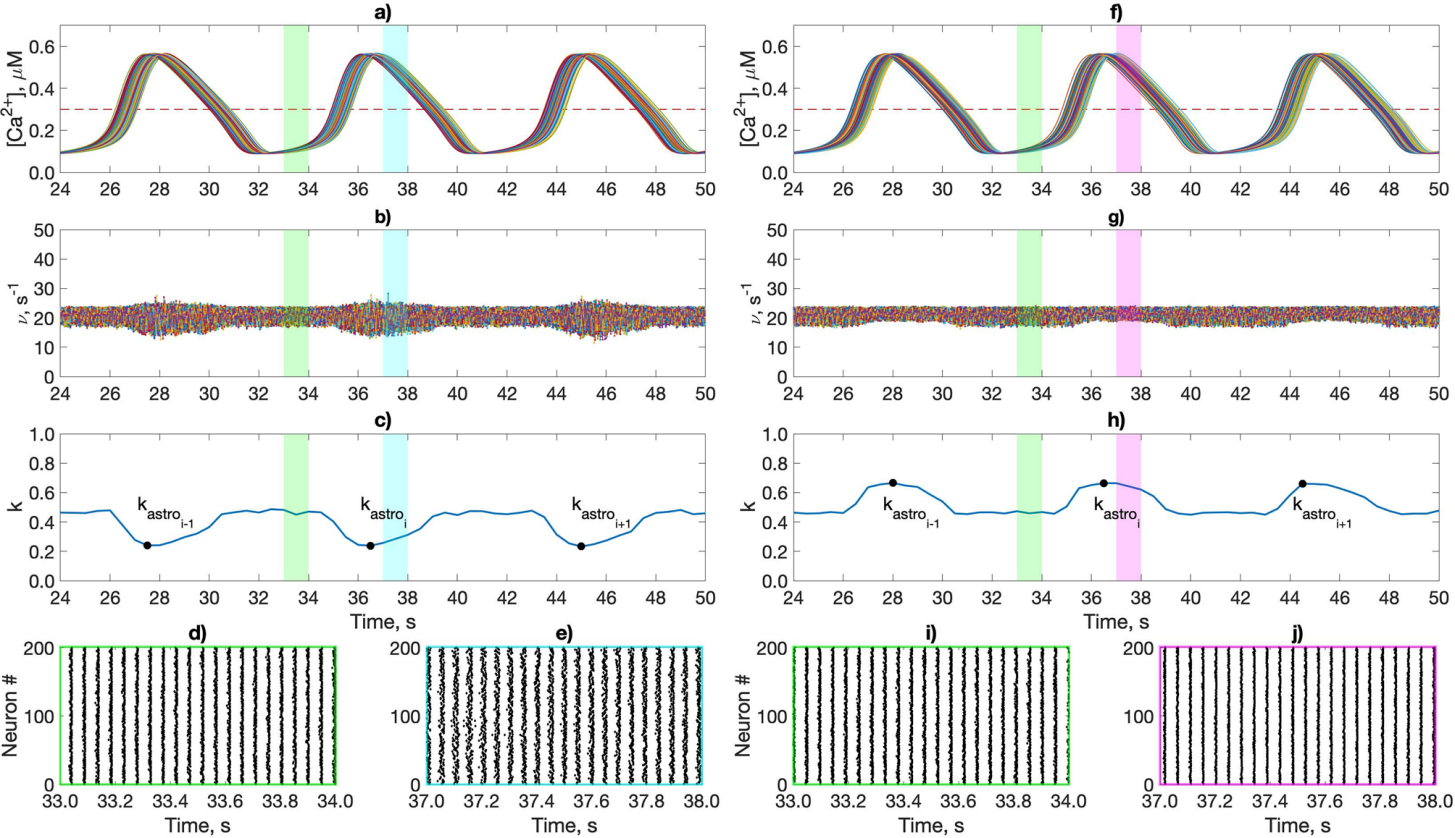
The astrocytic calcium dynamics induced by the activity of pyramidal neurons and its influence on $\gamma$ oscillations in the interneuron network through astrocyte-mediated modulation of the inhibitory synaptic weights. (**a**,**f**) The intracellular Ca$^{2+}$ concentration in the astrocytic network. (**b**,**g**) Instantaneous firing rates of interneurons. (**c**,**h**) Coherence network (*k*) dynamics. (**d**,**e**,**i**,**j**) Raster plots of an interneuron network activity. The dotted lines show the threshold Ca$^{2+}$ concentration for the astrocytic modulation of the synapse [Ca$^{2+}$] = 0.3 μM. Color coding of the 1 second long fragments of network activity: green—without astrocytic influence; blue—astrocyte-mediated depression of the inhibitory synapses ($g_{astro} = -\,0.8$); magenta—astrocyte-mediated facilitation of the inhibitory synapses ($g_{astro} = 1.2$). An example of used minimum/maximum values of the interneurons coherence during the Ca$^{2+}$ elevations in astrocytes marked with dots $k_{astro}$. $F_{in} = 260\ {\text{s}}^{-1}$; $g_{syn} = 0.009$ mS/cm^2^; $g_{syn_P} = 0.7$ mS/cm^2^.

**Figure 4:**
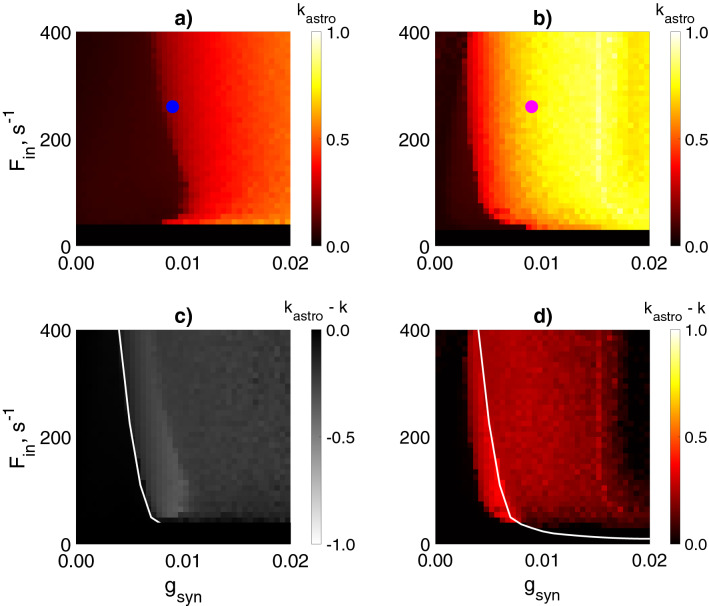
Influence of the astrocyte-induced modulation of the inhibitory synaptic transmission on the interneuron network coherence. Mean network coherence ($k_{astro}$) during astrocytic regulation of synaptic transmission is plotted against the frequency of excitatory inputs ($F_{in}$) and the synaptic weight between interneurons ($g_{syn}$) for astrocyte-mediated depression, $g_{astro} = -0.8$, (**a**) and facilitation, $g_{astro} = 1.2$, (**b**) of the inhibitory synapses. Filled circles indicate the parameter settings used in the simulations shown in Fig. 3. (**c**) Difference between (**a**) and Fig. 2c. (**d**) Difference between (**b**) and Fig. 2c. The white line corresponds to the border of the coherence region in the interneuron network without astrocyte (Fig. 2c). Each pixel represents an average of over 10 simulations. $g_{syn_P} = 0.7$ mS/cm^2^.

The dynamics of interneuron network model used in this study has been investigated in numerous previous studies^9,11,12,17^. In this study, we focused on the variation of two parameters of the astrocyte-induced regulatory action: the direction and the magnitude of the synaptic change. Without the astrocytes, an interneuron network in default conditions^11,12,17^ displays coherent fast oscillations with $k=0.5$ and the frequency of 21 Hz (Fig. 2a,b) which corresponds to the lower bound of the gamma-frequency range observed in the hippocampal interneuronal networks during behavioral arousal^11^. The interneuronal activity was induced by the stochastic excitatory synaptic inputs from pyramidal neurons driven by Poisson trains with a given frequency, $F_{in}$, chosen in accordance with earlier estimates. The emergence of interneuron synchronization and network coherence estimated by *k* is critically dependent on the frequency of excitatory inputs ($F_{in}$) and the unitary inhibitory synaptic weight ($g_{syn}$). We analyzed the dependence of coherence level in the interneuron network signaling on $g_{syn}$ and $F_{in}$ (Fig. 2c). In the selected range of parameter values, the network synchrony is realized when $F_{in}$ is larger than the critical value $F_{in\ min}$, which is required for signal transmission from pyramidal neurons to interneurons. $F_{in\ min}$ nonlinearly decreases with the enhancement of inhibitory synapses till $g_{syn}\approx 0.01$ mS/cm^2^. The coherence of interneuron network oscillations increases with synaptic strength.

### Influence of the astrocytic modulation of the inhibitory synaptic transmission on $\gamma$ oscillations in the interneuron network

We further explored the impact of the astrocytic modulation of synaptic transmission between interneurons on the coherence (*k*) of network oscillations. Two cases were considered: astrocyte-induced depression and potentiation of inhibitory transmission. Spiking activity of pyramidal neurons leads to the emergence of the calcium oscillations in astrocytes, which originally remained in the steady state (Fig. 3a,f). The dynamics of $\gamma$ rhythm modulation mediated by astrocytes is shown in Fig. 3. During astrocytic facilitation of the inhibitory synapses, the network oscillated with markedly increased coherence (Fig. 3g–j), while astrocyte-induced depression of coupling between interneurons induces coherence decrease (Fig. 3b–e).

To investigate the phenomenon in more detail, we explored network oscillations coherence at the intervals of astrocytic modulation (when [Ca$^{2+}$] > 0.3 μM). The maximum (for astrocyte-induced potentiation of inhibitory transmission) or minimum (for astrocyte-induced depression of inhibitory transmission) of the coherence coefficient were calculated ($k_{astro_i}$ on Fig. 3c,h), which were then averaged over all calcium pulses in the simulation. Next, we examined the dependence of the network coherence during astrocyte-mediated modulation, $k_{astro}$, on $g_{syn}$ and $F_{in}$ (Fig. 4a,b) and compared it with the coherence of interneuron activity without astrocytes, *k* (Fig. 2c).

Astrocyte-induced reduction of synaptic strength results in the decrease of $\gamma$ oscillations coherence in the interneuron network in all considered ranges of parameters $g_{syn}$, $F_{in}$ (Fig. 4a,c). Therefore, reaching a high level ($k>0.6$) of coherence for considered small values of synaptic weights $g_{syn}<0.02$ mS/cm^2^ becomes impossible.

On the contrary, the interneuron network with astrocyte-mediated potentiation of inhibitory transmission oscillates with markedly increased coherence. Such astrocytic impact induces the extension of the high-coherence region and leads to its shift to lower $g_{syn}$ value (0.016 mS/cm^2^ < $g_{syn}$ < 0.02 mS/cm^2^  in the absence of astrocytes versus 0.008 mS/cm^2^ < $g_{syn}$ < 0.02 mS/cm^2^ for astrocytic enhancement of the synaptic strength (Fig. 4b,d). The astrocytic influence extends the region of coherent oscillations to the range of weaker synaptic conductances, which belong to the experimentally determined postsynaptic conductances range^15^. The parameter dependence curve ($g_{syn}$, $F_{in}$) determines the occurrence of synchronization shifts to lower values of $g_{syn}$ for the entire range of $F_{in}>F_{in\ min}$ (Fig. 4b,d).

### Influence of the astrocytic modulation of the excitatory synaptic inputs on $\gamma$ oscillations in the interneuron network

Experimental studies showed that the tonic excitatory input currents in interneurons that induce $\gamma$ oscillations are small and highly heterogeneous^20,21^. However, previous interneuron network models required a highly homogeneous tonic excitatory drive or strong synaptic conductance values to compensate for the increased level of heterogeneity for emergence of synchronized activity^12,15,69^. Search for mechanisms to improve the robustness of generation of highly coherent gamma oscillations in the interneuron network has remained a relevant task for over two decades^17^. We, therefore, investigated how astrocytic modulation of excitatory synaptic drive from pyramidal neurons to interneurons can contribute to $\gamma$ rhythm formation.

**Figure 5:**
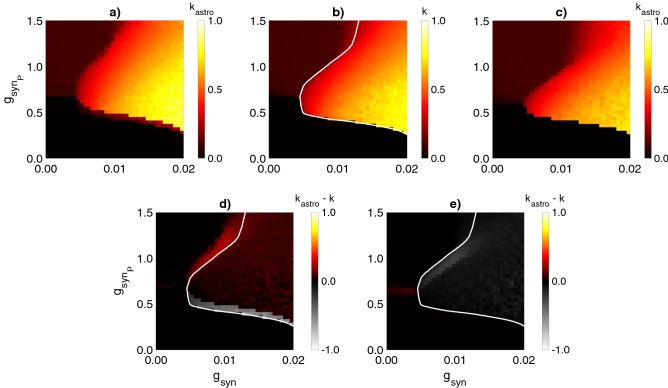
Influence of the astrocyte-induced modulation of the excitatory synaptic transmission on the interneuron network coherence. Mean network coherence, *k*, without astrocytic regulation (**b**) and during astrocytic regulation of synaptic transmission, $k_{astro}$, (**a**,**c**) is plotted against the strength of excitatory synaptic transmission ($g_{syn_P}$) and the synaptic weight between interneurons ($g_{syn}$). Astrocyte-mediated depression, $g_{astro} = -\,0.3$, (**a**) and facilitation, $g_{astro} = 0.3$, (**c**) of the excitatory synapses. (**d**) Difference between (**a**) and (**b**). (**e**) Difference between (**c**) and (**b**). The white line corresponds to the border of the coherence region in the interneuron network without astrocytes (*k* = 0.4). Each pixel represents an average of over 10 simulations.

In the model, the amplitudes of the excitatory synaptic currents in interneurons are determined by the strength of synaptic transmission between pyramidal neurons and interneurons, $g_{syn_P}$. In accordance with experimental data^9,17^, each pyramidal neuron was activated by Poisson pulse trains at a mean frequency of $F_{in} =$ 260 ${\text{s}}^{-1}$ with amplitudes chosen randomly from a uniform distribution within a given range [0.0; 2.5] μA/cm^2^. Without astrocytes for a fixed frequency, $F_{in}$, the synchronization emerges only within a specific subregion of the parameter space ($g_{syn}, g_{syn_P}$) (Fig. 5b), which expands as the strength of inhibitory coupling between interneurons, $g_{syn}$ increases. The lower boundary of the coherence region, $g_{syn_P\ min}$, corresponds to the minimum amplitude of the excitatory drive required for activity generation in interneurons (action-potential threshold). $g_{syn_P\ min}$ decreases nonlinearly as the interneuron coupling strength rises. The high-coherence ($k>0.6$) network oscillations almost reaching the threshold values $g_{syn_P}$ were observed in the model with strong inhibitory connectivity.

**Figure 6:**
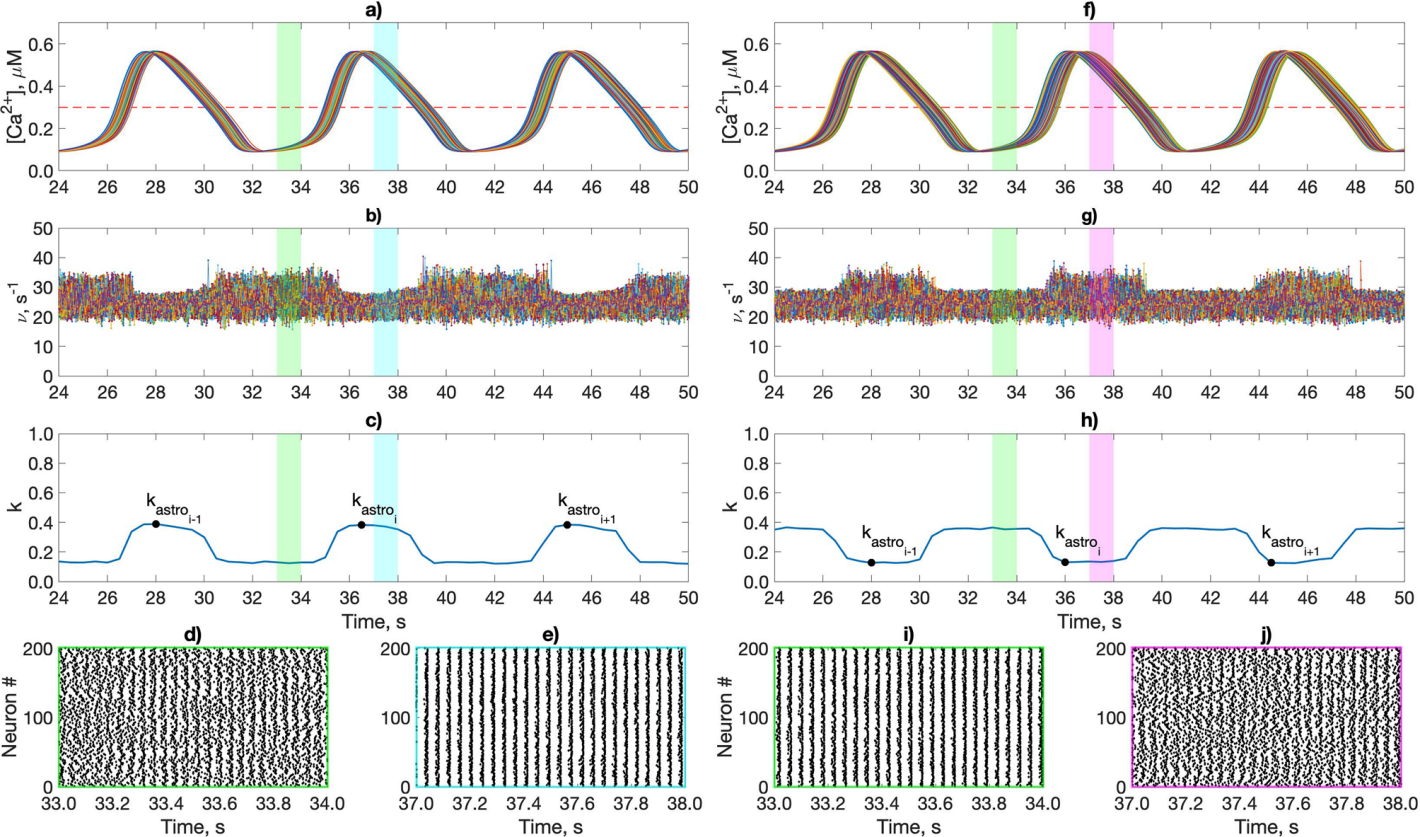
Dynamics of the astrocytic influence on $\gamma$ oscillations in the interneuron network through regulation of the excitatory synaptic drive. (**a**,**f**) The intracellular Ca$^{2+}$ concentration in the astrocytic network. (**b**,**g**) Instantaneous firing rates of interneurons. (**c**,**h**) Coherence network (*k*) dynamics. (**d**,**e**,**i**,**j**) Raster plots of an interneuron network activity. The dotted lines show the threshold Ca$^{2+}$ concentration for the astrocytic modulation of the synapse [Ca$^{2+}$] = 0.3 μM. Color coding of the 1 second long fragments of network activity: green—without astrocytic influence; blue—astrocyte-mediated depression of the excitatory synaptic transmission ($g_{astro} = -0.4; g_{syn_P} = 1.2$ mS/cm^2^); magenta—astrocyte-mediated facilitation of the excitatory synaptic transmission ($g_{astro} = 0.4; g_{syn_P} = 0.8$ mS/cm^2^). Minimum/maximum values of the interneurons coherence during the Ca$^{2+}$ elevations in astrocytes are marked with dots $k_{astro}$. $F_{in} = 260\ {\text{s}}^{-1}$; $g_{syn} = 0.01$ mS/cm^2^.

Similar to the previous scenario with astrocytic modulation of the inhibitory synaptic transmission, we analyzed the contribution of the gliotransmitter-induced potentiation and depression of the excitatory synapses to the modulation of the interneuron network coherence. The astrocytic suppression of excitatory synaptic drive ($g_{astro}<0$) evokes the significant network coherence enhancement (Fig. 6a–e). During astrocytic depression of the excitatory synaptic transmission, the high-coherence region expanded considerably, while the entire region of coherence slightly shifted to higher $g_{syn_P}$ values (Fig. 5a,d). Conversely, when astrocytes facilitated the synaptic drive ($g_{astro}>0$), the network oscillated with low coherence (Fig. 6f–j). Astrocyte-enhanced amplitudes of the excitatory synaptic currents result in high heterogeneity of the intrinsic firing rates of individual interneurons. Thus, the global network synchrony maintenance requires stronger connectivity between interneurons (Fig. 5c,e).

**Figure 7:**
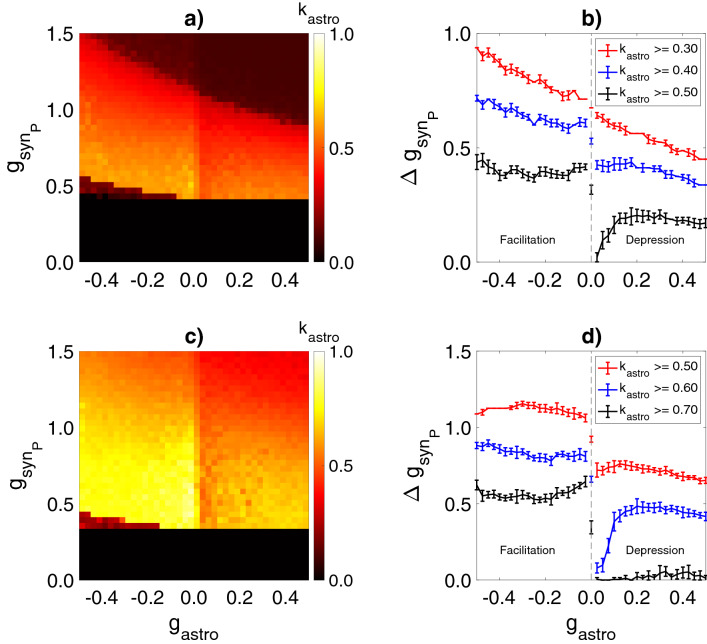
(**a**,**c**) Mean network coherence during the astrocytic regulation of synaptic transmission, $k_{astro}$, is plotted against the strength of the excitatory synaptic transmission ($g_{syn_P}$) and the magnitude of astrocytic modulation of the excitatory synaptic weights ($g_{astro}$). (**b**,**d**) Dependence of the $g_{syn_P}$ value range corresponding to the different coherence levels on the astrocytic modulation strength, $g_{astro}$. Each point represents an average ± standard deviation over 10 simulations. (**a**,**b**) $g_{syn}=0.01$ mS/cm^2^, (**c**,**d**) $g_{syn}=0.017$ mS/cm^2^.

Next, we analyzed the dependence of coherence on the astrocytic modulation ($g_{astro}$) and the excitatory synaptic strength ($g_{syn_P}$). Two values of interneuronal connectivity ($g_{syn}$) were compared, 0.01 mS/cm^2^ corresponded to medium coherence with $k \leqslant 0.5$, and 0.017 mS/cm^2^ corresponded to high coherence with $k \geqslant 0.5$ (Fig. 7a,c). We also examined how the $g_{syn_P}$ range corresponding to different coherence levels depends on the astrocytic modulation strength, $g_{astro}$ (Fig. 7b,d).

Astrocytic depression gain leads to considerable extension of $g_{syn_P}$ range corresponding to the medium-coherence region and minor variation of the high-coherence region. In this case, the lower bound $g_{syn_P\ min}$ of the coherence region slightly shifts to higher $g_{syn_P}$ values. Astrocyte-mediated facilitation of excitatory synaptic drive results in a drastic decrease of $g_{syn_P}$ range for the medium- and, especially, for the high-coherence regions. Further enhancement of this astrocytic influence leads to the narrowing of coherence regions. Wherein the critical value $g_{syn_P\ min}$ remained constant (Fig. 7).

Thus, pyramidal cells-astrocytes interaction can markedly enhance the coherence of oscillations in the interneuron network through the gliotransmitter-mediated decrease of excitatory synaptic drive heterogeneity. Astrocytic modulation of excitatory synaptic input can essentially improve the robustness of interneuron network $\gamma$ oscillations induced by physiologically relevant low and heterogeneous excitatory drive. In turn, astrocyte-induced potentiation of the excitatory synaptic transmission can lead to the reduction of oscillations coherence and can contribute to the impairment of $\gamma$ rhythm formation.

## Influence of the astrocytic modulation of synaptic transmission on gamma rhythm frequency in the interneuron network

**Figure 8:**
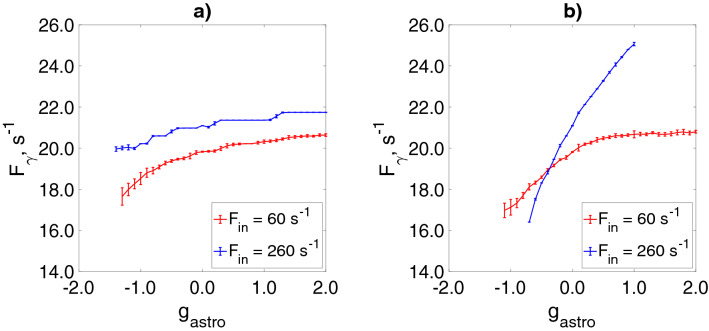
Dependencies of the network oscillations frequency on the astrocytic modulation strength, $g_{astro}$ for the astrocyte-mediated regulation of the inhibitory synaptic transmission (**a**) and of the excitatory synaptic transmission (**b**) for two different values of $F_{in}$.

The mechanisms contributing to the control of interneuron network gamma oscillations frequency are poorly understood. It is believed that they are critically dependent on the dynamics of the inhibitory synaptic conductance^7,10,11^. Computational studies indicate that frequency is regulated over a wider range by synaptic properties, tonic excitatory drive, excitation-inhibition balance, network structure, and electrical coupling^15–17^. To understand how the astrocytic modulation of synaptic transmission affects the network oscillations frequency, for two considered scenarios of neuron-astrocytic interaction, we examined the dependence of gamma oscillations frequency on the direction and magnitude of astrocytic influence (Fig. 8). The frequency of network oscillations, $F_{\gamma }$, was calculated only for the neural network activity with coherence $k>0.2$, otherwise $F_{\gamma }=0$. Astrocytic regulation of the inhibitory synaptic transmission in the interneuron network had only minimal effects on the network frequency (Fig. 8a). Astrocytic facilitation of the excitatory synaptic inputs to the interneurons results in a slight increase of the network oscillations frequency shifts it to the low $\gamma$ band. (Fig. 8b). Thus, astrocytic modulation of the synaptic transmission in the proposed model does not control the network frequency.

## Discussion

In this study, we investigated the influence of the astrocytic modulation of synaptic transmission on the generation of synchronized oscillations in the interneuron network. Recent experimental evidence that astrocytes contribute to the cortical gamma oscillations and recognition memory^23,24^ prompted us to develop a theoretical framework, which allowed us to investigate dynamical mechanisms of interplay between slow modulatory astrocytic signaling and fast synaptic transmission in formation of coherent $\gamma$ oscillations in the interneuron network. The proposed neuron-astrocyte network model incorporates the experimentally confirmed effects of astrocyte-mediated potentiation and inhibition of synaptic connection in a classical neural network model involving hippocampal interneurons (BCs) and principal neurons (pyramidal cells). The model describes the interaction of neurons and astrocytes on a network scale, at which groups of astrocytes activated by neurons form a functional dynamic network (via gap junction connectivity), and regulate the signaling of the neuronal network via the activity of gliotransmitters on synaptic connections. We found that the astrocytic regulation of signal transmission between neurons increases firing synchronicity and extends the region of coherent oscillations in biologically relevant values of the synaptic strengths. In particular, we showed that astrocyte-mediated potentiation of inhibitory synaptic transmission markedly enhances the coherence of network oscillations over a broad range of model parameters and leads to emergence of the synchronization in the interneuron network with weaker synapses. Gliotransmitter-induced depression of synaptic transmission between pyramidal cells and interneurons improves the robustness of the interneuron network gamma oscillations induced by physiologically relevant low and heterogeneous excitatory drive.

According to the in vitro experimental studies of gamma oscillations formation^10,20^, tonic excitatory currents in interneurons through metabotropic glutamate receptors and kainate receptors have small amplitudes with coefficient of variation from 35$\%$^21^ to 53$\%$^20^. Models with slow and weak synaptic connections between interneurons are able to generate oscillatory activity if heterogeneity in tonic excitatory drive is less than 3$\%$^11^. In interneuron network models with fast and high inhibitory conductance, coherence can be achieved for the tonic excitatory drive with large amplitude and heterogeneity levels not more than 10$\%$^12,15,22,69^. When shunting inhibition is incorporated, coherent oscillations ($k \geqslant 0.15$) can be induced by the excitatory current with low amplitude (0.5 μA/cm^2^) and high heterogeneity levels (up to 70$\%$)^17^. Under these conditions, the high-level coherence ($k \geqslant 0.5$) can be achieved for heterogeneity levels below 25$\%$ with high $g_{syn}$ values ($g_{syn} > 0.1\ {\text{mS}}/{\text{cm}}^2$). Moreover, if Poisson trains of fast excitatory synaptic conductances with biologically relevant kinetic properties^70^ are used instead of tonic excitatory currents similar to our study, the network coherence level drops below 0.4 ($k<0.4$) for moderate heterogeneity level 10$\%$ and for $g_{syn} > 0.1\ {\text{mS}}/{\text{cm}}^2$^17^. In our model, pyramidal neuron stimulation with Poisson trains with coefficient of variation $\sim$ 57$\%$ for pulse amplitudes leads to the formation of heterogeneous excitatory drive received by interneuron network. These excitatory currents characterized by cell-to-cell heterogeneity levels from 25$\%$ to 45$\%$ (determined by value of $g_{syn_P}$) and low mean amplitudes (less than 0.15 $\upmu{\text{A}}/{\text{cm}}^2$), consistent with experimental data. Here, we show that astrocyte-induced modulation of synaptic transmission helps to achieve high-level coherence ($k \geqslant 0.5$) with low $g_{syn}$ values ($g_{syn} < 0.02\ {\text{mS}}/{\text{cm}}^2$) for such small and highly variable excitatory drive (Figs. 4b,d, 5a,d).

Although bidirectional neuron-astrocyte interaction occurs on significantly slower temporal scales (seconds to minutes) in contrast with timescales of neuronal firing and excitatory or inhibitory synaptic transmission (milliseconds), the results of this study demonstrated that astrocytes can strongly regulate the fast dynamics of neural circuits that underlie normal cognitive behavior.

Recent experimental studies provided the lines of evidence that support multifunctional astrocytic contribution to local synaptic plasticity and coordination of neural network oscillatory activity, which in turn influence the information processing (for review see^31^). Notably, both inhibitory and stimulatory effects of astrocytic modulation on the gamma oscillations are reported in the hippocampus, which is in line with the previously discussed simulation results. The optogenetic activation of ChR2-expressing astrocytes reduces the power of kainate-induced hippocampal ex vivo gamma oscillations via regulation of pyramidal cell and interneuron excitability by astrocyte-released ATP and/or adenosine^25^. The positive astrocytic influence on the gamma rhythm formation was observed in mice with genetically induced suppression of astrocytic exocytosis, which showed reduced electroencephalographic (EEG) power spectrum in the gamma frequency range in vivo and impairment of carbachol-induced gamma oscillations in vitro^23^. The activation of the astrocytic GABA$_B$ signaling triggers gliotransmission, which regulates the oscillatory activity of the neuronal network and increases the theta and gamma EEG power spectrum in vivo in mice^24^. Several studies provided direct evidence of the involvement of astrocyte signaling in cognitive functions and behavior^33,71^. However, in many of the cases, the understanding of astrocytic involvement is still incomplete. Now that we better understand the molecular components of astrocyte-neuron interactions, the new challenge is to investigate how they integrate at the network and physiological levels. To address this question, novel, bio-inspired, and detailed computational models should be developed to simulate the information processing through the coordinated activity of both astrocytes and neurons. Astrocytes provide an extradimensional influence over neuronal networks by a multiscale spatiotemporal integration of neural activity and can produce higher-order organization of the information coding^45^. The astrocytic Ca$^{2+}$ activity patterns could represent a guiding template that modifies the state of the local neuronal network. This modification can result in an intriguing possibility of a considerable increase of the information-possessing capacity of the mammalian brain, which could exceed the level we currently hypothesize^26^.

Various computational models of the neuron-astrocyte interaction on the network level have been successfully applied to study the specific effects of neuronal activity regulation by astrocytes (for a review, see^72^). For example, Kanakov and colleagues^42^ and later Abrego et al.^44^ investigated the role of astrocyte-induced modulation of synaptic transmission in information processing in small neuron-astrocyte ensembles. These studies showed that the astrocytes can induce formation of the spatiotemporal activity patterns in neural network due to astrocyte-mediated facilitation of the synaptic connectivity at the timescale of astrocytic dynamics. Lenk and colleagues^35^ studied the astrocytic contribution to the maintenance of the average neuronal activity level. This study showed that astrocytes could promote homeostatic regulation of the firing rate in the neuronal network similar to the influence of the extracellular matrix^73^; astrocyte-mediated modulation of synapses induces the stabilization of the neuronal activity, preventing neuronal hyperactivation. Additionally, Makovkin et al.^34^ presented a two-layer oscillatory network mimics the interconnected astrocytic and neuronal networks. The astrocytic layer consists of low frequency phase oscillators coupled locally. The neuronal layer employs high frequency oscillators interconnected non-locally with regular or random topology. The analysis of the mixed coupling role in the collective dynamics and synchronization formation in such a multiplex neuron-astrocyte ensemble demonstrated that the inhibitory connections in the neural subnetwork decreases the level of phase synchronization, but sufficiently strong coupling to the astrocytes recovers synchrony in the entire network.

Integration of astrocytic signaling in cognitive processing has implications for understanding the basis of cognitive alterations in pathological conditions. The abnormal astrocytic signaling can induce synaptic and network dysregulations leading to cognitive impairment^74–77^. Understanding the emerging role of astrocytes in regulating neural network oscillations underlying cognitive function and dysfunction opens a way for the development of novel pharmacological treatments for brain disorders.

## Supplementary Information

Supplementary Information 1.

## Data Availability

The code is available at https://github.com/SpaceQuester/HodgkinHuxleyUllahJung.
