# Supplementary material for: Controlling synchronization of gamma oscillations by astrocytic modulation in a model hippocampal neural network
Source: Sci Rep. 2022 Apr 28;12:6970. doi: 10.1038/s41598-022-10649-3 (PMC9050920; doi:10.1038/s41598-022-10649-3)
Supplement: Supplementary file 1 — Supplementary Information 1. [file 41598_2022_10649_MOESM1_ESM.pdf]

Supplementary Information

Appendix A

Dynamics of neuron membrane potential:

$$
\begin{aligned}
\alpha_m &= \frac{0.182(V_i+35)}{1-e^{\frac{-(V_i+35)}{9}}};\\
\beta_m &= \frac{-0.124(V_i+35)}{1-e^{\frac{(V_i+35)}{9}}};\\
\alpha_n &= \frac{0.02(V_i-25)}{1-e^{\frac{-(V_i-25)}{9}}};\\
\beta_n &= \frac{-0.002(V_i-25)}{1-e^{\frac{(V_i-25)}{9}}};\\
\alpha_h &= 0.25e^{\frac{-(V_i+90)}{12}};\\
\beta_h &= 0.25\frac{e^{\frac{V_i+62}{6}}}{e^{\frac{V_i+90}{12}}}.
\end{aligned}
\tag{1}
$$

Dynamics of intracellular calcium concentration in astrocytes:

$$
\begin{aligned}
&J_{channel\,i} = c_1 v_1 IP3_i^3 Ca_i^3 z_i^3 \left(\frac{c_0}{c_1} - (1+\frac{1}{c_1})Ca_i\right)/\\
&/\left[(IP3_i+d_1)(Ca_i+d_5)\right]^3;\\
&J_{pump\,i} = v_3 Ca_i^2/(k_3^2+Ca_i^2);\\
&J_{leak\,i} = c_1 v_2 \left(\frac{c_0}{c_1} - (1+\frac{1}{c_1})Ca_i\right);\\
&J_{in\,i} = v_5 + v_6 IP3_i^2/(k_2^2+IP3_i^2);\\
&J_{out\,i} = k_1 Ca_i;\\
&J_{PLC\,i} = v_4\left(Ca_i+(1-\alpha)k_4\right)/(Ca_i+k_4);\\
&J_{Cadiff\,i} = d_{Ca}(Ca_{i-1}+Ca_{i+1}-2Ca_i);\\
&J_{IP3diff\,i} = d_{IP3}(IP3_{i-1}+IP3_{i+1}-2IP3_i).
\end{aligned}
\tag{2}
$$

Appendix B

Neuron parameters: $C = 1\mu F$; $g_{Na} = 40\ mS/cm^2$; $g_K = 35\ mS/cm^2$; $g_{Leak} = 0.3\ mS/cm^2$; $E_{Na} = 55\ mV$; $E_K = -77\ mV$; $E_{Leak} = -54.4\ mV$; $E_{syn_i} = -90\ mV$; $k_{syn} = 0.2\ mV$; $I_{app_i} = 0.7\ \mu A/cm^2$; $\mu = 1000$.

Initial conditions for Stable Focus neurons: $V_i(t=0) = -58.7085$; $m_i(t=0) = 0.0953$; $n_i(t=0) = 0.000913$; $h_i(t=0) = 0.3662$.

Initial conditions for Stable Limit Circle neurons: $V_i(t=0) = 14.8409$; $m_i(t=0) = 0.9174$; $n_i(t=0) = 0.0140$; $h_i(t=0) = 0.0539$.

Astrocyte parameters: $c_0 = 2\ \mu M$; $c_1 = 0.185$; $v_1 = 6\ s^{-1}$; $v_2 = 0.11\ s^{-1}$; $v_3 = 2.2\ \mu M/s$; $v_5 = 0.025\ \mu M/s$; $v_6 = 0.2\ \mu M/s$; $k_1 = 0.5\ s^{-1}$; $k_2 = 1\ \mu M$; $k_3 = 0.1$; $k_4 = 1.1\ \mu M/s$; $a_2 = 0.14\ \mu M/s$; $d_1 = 0.13\ \mu M$; $d_2 = 1.049\ \mu M$; $d_3 = 0.9434\ \mu M$; $d_5 = 0.082\ \mu M$; $\alpha = 0.8$; $\tau_{IP3} = 7.143\ s$; $IP3^* = 0.16\ \mu M$; $d_{Ca} = 0.001\ s^{-1}$; $d_{IP3} = 0.12\ s^{-1}$; $\alpha_{Glu} = 2$. We rescale the time units of the astrocyte model in order to match it in milliseconds for numerical integration.

Initial conditions for astrocytes: $Ca_i(t=0) = 0.07$; $IP3_i(t=0) = 0.16$; $z_i(t=0) = 0.67$; $G_i(t=0) = 0.0$.
